# Association of Parental Overweight and Cardiometabolic Diseases and Pediatric Adiposity and Lifestyle Factors with Cardiovascular Risk Factor Clustering in Adolescents

**DOI:** 10.3390/nu8090567

**Published:** 2016-09-13

**Authors:** Chun-Ying Lee, Wei-Ting Lin, Sharon Tsai, Yu-Chan Hung, Pei-Wen Wu, Yu-Cheng Yang, Te-Fu Chan, Hsiao-Ling Huang, Yao-Lin Weng, Yu-Wen Chiu, Chia-Tsuan Huang, Chien-Hung Lee

**Affiliations:** 1 Department of Public Health, College of Health Sciences, Kaohsiung Medical University, Kaohsiung 807, Taiwan; cying@ms19.hinet.net (C.-Y.L.); ych0610@gmail.com (Y.-C.H.); catstar1211@gmail.com (P.-W.W.); l19920929@gmail.com (Y.-C.Y.); 2 Department of Family Medicine, Kaohsiung Medical University Hospital, Kaohsiung 807, Taiwan; chtshu@cc.kmu.edu.tw; 3 Institute of Environmental and Occupational Health Sciences, National Yang Ming University, Taipei 112, Taiwan; wtlin0123@gmail.com; 4 Department of Laboratory Medicine, Kaohsiung Municipal Hsiao-Kang Hospital, Kaohsiung 807, Taiwan; 0870718@kmhk.org.tw; 5 Department of Obstetrics and Gynecology, Graduate Institute of Medicine, College of Medicine, Kaohsiung Medical University, Kaohsiung 807, Taiwan; tefu.chan@msa.hinet.net; 6 Department of Obstetrics and Gynecology, Kaohsiung Medical University Hospital, Kaohsiung Medical University, Kaohsiung 807, Taiwan; 7 Department of Oral Hygiene, College of Dental Medicine, Kaohsiung Medical University, Kaohsiung 807, Taiwan; hhuang@kmu.edu.tw; 8 Department of Food and Nutrition, Providence University, Taichung 433, Taiwan; ylweng@pu.edu.tw; 9 Health Policy and Systems Management Program, Health Sciences Center, School of Public Health, Louisiana State University, New Orleans, LA 70803, USA; ychiu@lsuhsc.edu; 10 Research Center for Environmental Medicine, Kaohsiung Medical University, No. 100 Shih-Chuan 1st Road, Kaohsiung 807, Taiwan

**Keywords:** adolescent, adiposity, cardiometabolic risk factors, metabolic syndrome, parental disease, screen time, sugar-sweetened beverages

## Abstract

Cardiometabolic risk factors or their precursors are observed in childhood and may continue into adulthood. We investigated the effects of parental overweight and cardiometabolic diseases and pediatric lifestyle factors on the clustering of cardiovascular risk factors among adolescents, and examined the mediating and modifying effects of pediatric adiposity on these associations. Representative adolescents (*n* = 2727; age, 12–16 years) were randomly recruited through multistage stratified sampling from 36 schools in Southern Taiwan. Adolescent and parent surveys were conducted in schools and participant homes, respectively. Their demographic factors, diet patterns, and physical, anthropometric, and clinical parameters were collected and analyzed. Adolescents with 1–2 and ≥3 risk components for pediatric metabolic syndrome (MetS) were defined as potential MetS (pot-MetS) and MetS, respectively. Adolescents whose parents were overweight/obese, or with diabetes and hypertension had a higher prevalence ratio of pot-MetS and MetS (1.5–1.6 and 1.9–4.2-fold, respectively). Low physical activity (<952.4 MET·min/week), long screen time (≥3 h/day) and high sugar-sweetened beverage intake (>500 mL/day) were associated with a 3.3- (95% confidence intervals (CI) = 1.5–7.3), 2.2- (95% CI = 1.1–4.4), and 26.9-fold (95% CI = 3.2–229.0) odds ratio (OR) of MetS, respectively. Pediatric body mass index (BMI) accounted for 18.8%–95.6% and 16.9%–60.3% increased prevalence ratios of these parental and pediatric risk factors for MetS. The OR of pot-MetS + MetS for sugar-sweetened beverage consumption was multiplicatively enhanced among adolescents with overweight/obesity (combined OR, 8.6-fold (95% CI = 4.3–17.3); *p* for multiplicative interaction, 0.009). The results suggest that parental overweight and cardiometabolic diseases and pediatric sedentary and high sugar-intake lifestyles correlate with the development of adolescent MetS, and an elevated child BMI explains a part of these associations. Pediatric adiposity might be multiplicatively associated with sugar-sweetened beverage consumption for enhancing the MetS prevalence ratio among adolescents.

## 1. Introduction

Type 2 diabetes mellitus (T2DM) and its long-term complications, including cardiovascular diseases, stroke, and chronic kidney failure, are global health concerns that substantially affect life expectancy and mortality [1]. In adults, principal determinants, such as abdominal obesity, dyslipidemia, hypertension, and dysglycemia, and the clustering of these disorders have been reported [2]. Nevertheless, some of these risk factors or their precursors are observed in childhood [3,4,5], and may persist into adulthood [6]. Metabolic syndrome (MetS) is a cluster of cardiometabolic risk factors. A longitudinal study revealed that pediatric MetS predisposes adults to a high risk of high carotid intima-media thickness and T2DM [7]; however, the risk can be reduced to normal by resolving MetS before adulthood [8]. These findings emphasize that preventing and resolving pediatric MetS is crucial for preventing the development of these cardiometabolic disorders in adulthood.

Parent–child dyad studies have suggested that obesity and certain cardiometabolic risk factors are shared across generations [9,10,11]. A study investigating longitudinal cardiovascular risk found that obese children with an obese parent were 21.2% likely to be eligible for bariatric surgery in adulthood [12]. However, whether children with parental MetS-related diseases are at a high risk of MetS is uncertain. If such a risk exists, whether the generational effect is associated with pediatric adiposity remains to be investigated. Lifestyle risk factors, such as unhealthy diets, physical inactivity, and excessive screen time (ST), have been associated with increased cardiometabolic risk among children and adolescents [13,14,15,16,17]. MetS is common in obese youths [18], and numerous unhealthy behaviors have been identified as obesogenic risk factors [19,20,21]. Therefore, the mediating and/or modifying effects of obesity on the association between lifestyle risk factors and pediatric MetS warrant further investigation.

In Taiwan, childhood overweight and obesity have received extensive attention as major health concerns. According to a series of national surveys conducted by Taiwan’s Ministry of Education between 1991 and 2003, the prevalence of overweight/obesity among adolescents aged 13–18 years increased by 53.7% (from 16.4% to 25.2%) in boys and 70.8% in girls (from 8.9% to 15.2%) [22]. With the markedly high increase in childhood overweight and obesity, the clustering of cardiometabolic risk factors among adolescents has become a critical concern in pediatric health care. The present study had two aims: first, to evaluate the effects of parental overweight and MetS-related diseases in addition to pediatric lifestyle risk factors on different degrees of clustering of cardiovascular risk factors, and second, to investigate the mediating and modifying effects of pediatric adiposity on the association between these factors and cardiometabolic disorders among Taiwanese adolescents.

## 2. Materials and Methods

### 2.1. Participants

In this cross-sectional study, we used data from the Multilevel Risk Profiles for Adolescent Metabolic Syndrome study, a large-scale survey designed to obtain estimates on diet, physical activity (PA), and cardiometabolic disease-related risk markers from a representative adolescent population in Southern Taiwan [4,5,23,24]. The target population comprised adolescents who were included in the entry lists of 112 junior high schools’ grades 7–9 (age, 12–16 years) from 2007 to 2009 in Kaohsiung City, Pingtung County, and Taitung County of Southern Taiwan; these three areas have diverse economic levels. The complex sampling approach and research procedures have been detailed previously [4]. In brief, a multistage, geographically-stratified cluster sampling technique was used to enroll study participants from the defined population. Overall, 3881 students were randomly enrolled from 36 randomly selected schools. Among them, 3784 (97.5%) agreed to participate in the questionnaire survey and anthropometric measurements; 2727 (72.1%) of these students underwent biochemical blood examinations. The study was commenced after the Institutional Review Board of Kaohsiung Medical University approved the research protocol, and written informed assent and consent were respectively obtained from the adolescents and their parents or guardians.

### 2.2. Data Collection

Two structured questionnaires developed for adolescents and their parents were used to collect the research information. The adolescent survey was conducted in schools, where adolescent questionnaires were completed with the assistance of trained research staff and school teachers. We collected data on demographic characteristics, individual disease history, PA, sedentary lifestyle factors, dietary consumption, and the status of alcohol intake and cigarette smoking. The parent survey was conducted in participant homes, where a self-administered questionnaire with a clear fill description was completed. The data obtained comprised socioeconomic factors, body weight, height, disease history, dietary intake, PA, alcohol drinking and cigarette smoking. Body mass index (BMI) was determined by dividing the weight (kg) by the square of the height (m^2^). Normal weight was defined as a BMI of ≤23.9, overweight as 24 ≤ BMI < 26.9, and obesity as BMI ≥ 27 according to the adult criteria determined by Taiwan’s Department of Health [25]. Disease data included hypertension, T2DM, cardiovascular diseases, and other chronic diseases. A positive disease history was defined as either the father or mother having a clinically-diagnosed disease. 

A scale containing nine questions was used to measure the PA of adolescents on both weekdays and weekends. The activity data were converted to metabolic equivalent task (MET) measurements, and the overall MET·min per week was calculated as the product of the MET level of activity, time (minutes) spent in each activity per day, and the number of days per week [26]. Information on sedentary lifestyle factors included time spent on watching television, playing video games, working on a computer at home or in an internet cafe, reading books, and after-school tutoring. ST was defined as the time spent on activities performed in front of a screen; it was measured as the average time spent on watching television, playing video games, and using the computer per day in a typical week (including weekdays and weekends). Reading time was defined as the time spent on reading-related sedentary activities; it was measured as the average time spent on reading books and after-school tutoring per day in a typical week.

The daily dietary intake over the preceding one month was analyzed by using a semi-quantitative food-frequency questionnaire comprising 23 food groups. The Taiwan Food and Drug Administration established a Food and Nutrients Databank for foods often consumed by Taiwanese people [27]. We used this databank and individual dietary data to estimate the daily total calorie intake. Data on the consumption of soft drinks, sweetened teas, and fruit drinks were derived from the responses to the food-frequency questionnaire. Sugar-sweetened beverage (SSB) drinkers were defined as participants who had ingested at least one serving of these drinks per week in the previous month; the daily amount of SSB intake was calculated for each participant. A positive status for drinking or smoking was defined as participants who reported drinking ≥1 drinks of any types of alcoholic beverages or smoking ≥1 cigarettes in the preceding month.

### 2.3. Anthropometric and Clinical Measurements

Certified examiners performed anthropometric assessments in the adolescents to obtain data on height, weight, waist circumference, body fat, and blood pressure (BP). Because the adolescent BMI varies according to age and sex, we converted the BMI to age- and sex-standardized *Z*-scores on the basis of Taiwanese adolescent growth charts and presented the value as zBMI for data analysis. Bodyweight was classified according to adolescent BMI and the age- and sex-specific BMI cutoff points for adolescent overweight and obesity determined by Taiwan’s Ministry of Health and Welfare [28]. A mercury sphygmomanometer was used to measure blood pressure according to the American Heart Association guidelines [29]. Two blood pressure measurements were performed at five-minute intervals, and the average systolic and diastolic blood pressure readings were calculated for analysis. After collecting the questionnaire data, the researchers required three weeks to obtain informed consent from the parents of all participating students, following which the blood examinations were conducted. Clinical samples were obtained from the adolescents whose parents or guardians offered informed consent. Moreover, blood samples after a 10-h overnight fast were collected in the morning through venipuncture at the health center of each school. Overnight fasting was confirmed through face-to-face inquiries regarding the food ingested between the preceding night and the examination morning. Using commercially available reagents, we measured the circulating levels of high-density lipoprotein cholesterol (HDL-C), triglycerides, and fasting plasma glucose by using a chemistry autoanalyzer (TBA-c16000 automatic analyzer, Toshiba, Tokyo, Japan) [30].

### 2.4. Definition of Metabolic Syndrome

No universal diagnostic criteria exists for pediatric MetS. Therefore, to investigate its associated risk factors among adolescents, the harmonized criteria proposed by a joint statement from six obesity-, diabetes-, and heart-related major societies were used to define MetS [31]. The definition of MetS includes five risk components: (1) elevated fasting plasma glucose (≥100 mg/dL); (2) decreased HDL-C males, <40 mg/dL (1.0 mmol/L) and females, <50 mg/dL (1.3 mmol/L)); (3) elevated triglycerides ≥150 mg/dL (1.7 mmol/L)); (4) central obesity (waist circumference: ≥90 cm and ≥80 cm for Asian males and females, respectively); and (5) elevated BP (systolic BP, ≥130 mmHg and diastolic BP, ≥85 mmHg). Participants with ≥3 risk components were defined as having MetS. To investigate the effects of risk factors on the potential status of MetS (pot-MetS) in adolescents, we defined participants who have one or two risk components as pot-MetS. Participants without any risk components were defined as non-MetS risk factor (non-MetS) and were considered the control group.

### 2.5. Statistical Analysis

In the study areas, a high proportion of aboriginal people live in Taitung County (approximately 30% of the inhabitants). To investigate possible between-race differences in effects, Taitung’s respondents were oversampled to ensure sufficient adolescents in the diverse ethnicity groups. Survey-data modules were used to adjust for the multistage sampling design and unequal sampling probabilities (Stata v13, StataCorp., College Station, TX, USA), so as to final results can be representative of the student population of Southern Taiwan. The weighted results were expressed as means, standard errors, or percentages of variables. For data interpretation, certain continuous factors were analyzed at a categorical scale as follows: PA, <952.4, 952.4–2140.4, and ≥2140.5 MET·min/week (tertile); ST, <1.5, 1.5–2.9, and ≥3 h/day; reading time, <1.5, 1.5–2.9, and ≥3 h/day; SSB intake, non-intake, 1–500 and >500 mL/day. Because three MetS-associated outcomes (non-MetS, pot-MetS, and MetS) were defined, we used multinomial logistic regression models to determine the prevalence ratio of pot-MetS or MetS relative to the non-MetS [32]. The adjusted odds ratio (aOR) with its 95% confidence intervals (CIs), obtained by exponentiating the corresponding regression coefficient, was used to evaluate the association between the study variables and the specific MetS status after adjustment for the effects of potential confounding factors. Parameters that changed the effect of interest by >10% or had been previously established as confounders were considered the potential confounding variables [33]. These confounding factors were age, gender, ethnicity, residential area, total calorie intake, alcohol-drinking status, and cigarette-smoking status. The adjusted mean is a statistic calculated from the prediction of a fitted model at a fixed value of the investigated variable and the average values of the remaining covariates. This statistic was used to compare the mean difference in adolescent risk factors across MetS-associated conditions. Because child BMI substantially affects MetS, we evaluated the excess prevalence ratio of pot-MetS and MetS that is explained by child BMI for each risk factor by using the formula (aOR_1_ − aOR_2_)/(aOR_1_ − 1)—where aOR_1_ is the prevalence ratio obtained from the covariate-adjusted base model, aOR_2_ is the prevalence ratio after additionally adjusting for the child BMI, and aOR_1_ − 1 is the excess prevalence ratio of a risk factor [34]. Multivariable logistic regression models with two parameters and their cross-product terms were used to analyze the potential synergistic effect by using a multiplicative model. The synergism index (SI), recommended by Rothman, was calculated to assess the empirical deviation from the additive interaction relationship [35,36]. An SI value that significantly differed from the null suggests an additive interaction effect.

## 3. Results

After adjusting the complex sampling design, the prevalence of pot-MetS and MetS among adolescents in Southern Taiwan was 39.3% and 3.3%, respectively (Table 1). The MetS group had a higher percentage of males than did the non-MetS group (63.4% vs. 48.0%). No notable discrepancy was observed in the distribution of age, ethnicity, residential area, total calorie intake, alcohol drinking, and cigarette smoking across adolescents in the non-MetS, pot-MetS and MetS groups. Adolescents with a poor MetS condition tended to have decreased physical activity and an increased SSB intake, central obesity, zBMI value, waist circumference, BP, serum triglyceride, and plasma glucose and were likely to have a low HDL-C level. 

The association between parental risk factors and adolescent MetS status is shown in Table 2. Parental overweight/obesity was observed to be associated with a higher cardiometabolic prevalence ratio in adolescents (aOR = 1.5 for pot-MetS in both father and mother and 2.2 for MetS in the mother). In addition, both parents being overweight/obese predisposed adolescents to a 2.3- and 3.2-fold higher prevalence ratio of pot-MetS and MetS, respectively. Adolescents with parental diabetes and hypertension had a 1.7- and 1.5-fold aOR of pot-MetS and 5.1- and 2.7-fold aOR of MetS, respectively, compared with those whose parents had no disease history. The prevalence ratio of MetS was also higher than that of pot-MetS among adolescents with parental diabetes and hypertension (aOR ratio = 3.0 and 1.8, respectively; *p* < 0.03).

Figure S1 illustrates the distribution of overweight and obesity among adolescents with different MetS status. A considerable decrease in the proportions of normal weight, overweight and obesity was observed in adolescents without any metabolic abnormality, whereas the corresponding body weight proportions notably increased in adolescents with MetS. Adolescent overweight/obesity was associated with a 3.9- and 1461.2-fold elevated prevalence ratio of pot-MetS and MetS, respectively (Table 3). The adjusted mean of the BMI for adolescents with MetS was 7.4 kg/m^2^ higher than that for adolescents with pot-MetS.

Table 3 presents the association between adolescent lifestyle factors and their MetS conditions. Compared with adolescents with a high level of PA (≥2140.5 MET·min/week), those with moderate (952.4–2140.4 MET·min/week) and low (<952.4 MET·min/week) levels of PA had a 2.5- and 4.4-fold higher aOR of MetS, respectively. Long ST (≥3 h/day) was associated with a higher MetS prevalence ratio, with adolescents with MetS having a 0.30 h/day longer ST than did non-MetS adolescents. Adolescents who consumed heavy amounts of SSB (>500 mL/day) had a 1.7- and 30.2-fold higher aOR of pot-MetS and MetS, respectively. For moderate and low levels of PA, a heterogeneous prevalence ratio was identified between pot-MetS and MetS (aOR ratio, 2.5 and 3.8, respectively). Considering SSB intake, the prevalence ratio of MetS was higher than that of pot-MetS (aOR ratio, 13.0 and 17.7 for 1–500 and >500 mL/day of SSB intake, respectively), and adolescents with MetS had a 68.1 mL/day higher SSB intake than did those with pot-MetS.

The excess prevalence ratios of pot-MetS and MetS explained by the adolescent BMI for significant risk factors are shown in Table 4. The child BMI accounted for 66.9%–72.9% of the effect of parental overweight/obesity on pot-MetS, as well as for 23.5%–40.1% and 18.8%–50.5% of the association of parental T2DM and hypertension with pediatric pot-MetS and MetS, respectively. A high proportion of excess prevalence ratio of MetS (60.3%) associated with ≥3 h/day of ST was explained by the child BMI. For heavy SSB consumption (>500 mL/day), the child BMI separately accounted for 23.6% and 16.9% of the effect on pot-MetS and MetS. Although the prevalence ratios between pot-MetS and MetS were heterogeneous for parental T2DM, hypertension, and adolescent low PA (aOR ratio, 2.6, 1.8, and 2.6, respectively), more than 10% of the association was explained by the adolescent BMI (44.4%, 10.2%, and 41.7%, respectively).

Table 5 shows the synergistic effects of risk factors and adolescent body weight on pot-MetS and MetS. The prevalence ratio (aOR = 8.6) of pot-MetS + MetS in SSB consumers who were overweight/obese was remarkably higher than the expected combined prevalence ratio (aOR = 2.9) estimated using a multiplicative interaction model (*p* = 0.009). A significantly additive synergistic effect on pot-MetS + MetS was also observed among overweight/obesity adolescents with parental T2DM (SI = 4.2, 95% CI = 1.4–12.4) and who had ≥1.5 h/day of ST (SI = 1.8, 95% CI = 1.2–2.9). No significant interactions were found across the other risk factors for pot-MetS + MetS (Table S1).

## 4. Discussion

The present findings reveal the association between parental overweight, T2DM, and hypertension and pediatric adiposity and lifestyle factors with the clustering of cardiometabolic risk factors among adolescents. This study also revealed the mediating and modifying effects of pediatric overweight on the association of familial and individual factors with adolescent pot-MetS and MetS.

Family linkage studies have reported that cardiovascular risk factors, such as obesity and hyperlipidemia, incline to cluster within families [9,10,11]. In a longitudinal parent–child dyad study, a 14.2-fold higher risk of eligibility for bariatric surgery was observed among obese children with an obese parent compared with children with non-obese families [12]. This study further revealed that parental overweight/obesity may predispose children to a higher risk of pot-MetS (aOR = 1.5 in both father and mother) and MetS (aOR = 2.2 in the mother). Recently, cardiometabolic abnormalities among children have been associated with parental MetS-associated diseases [37,38]. In India, a higher risk of being overweight, low HDL-C, high BP, and glucose intolerance was observed in adolescents with parental T2DM [38]. In Iran, a 4.5-fold risk of MetS was observed in adolescents with both parents having MetS. Our study revealed that a parental history of T2DM and hypertension was associated with pediatric pot-MetS and MetS, and the risk of MetS was higher than that of pot-MetS (aOR ratio, 3.0 and 1.8, respectively) in adolescents whose parents had the two MetS-related diseases.

This study revealed that the adolescent BMI accounted for a majority of the effects of parental overweight/obesity on pediatric pot-MetS and MetS (66.9%–72.9% for pot-MetS in both parents and 95.6% for MetS in the mother). This emphasizes the potential effect of a shared obesogenic environment across generations. Although adolescent BMI also accounted for 18.8%–50.5% of the increased risks of parental T2DM and hypertension on pediatric MetS, the generational effect of the two MetS-associated diseases remained significant (zBMI-adjusted OR = 2.6 and 2.4, respectively; *p* < 0.03), implying the existence of non-obesity-mediated mechanisms. These results were consistent with those of a recent study, in which many cardiometabolic risk factors significantly correlated between parents and children even after adjustments for their BMIs [10]. Unhealthy lifestyle factors are the major determinants of obesity, diabetes, and cardiovascular diseases [20,39]. Considering these findings that reveal the clustering of cardiometabolic risk factors and diseases within families, establishing a family-based healthy lifestyle is crucial for preventing and resolving the clustering of MetS components within families. Our study identified a notable modifying effect of parental T2DM on the risk of pot-MetS + MetS for adolescent overweight/obesity by using an additive interaction model (combined aOR = 23.8). This indicates that overweight adolescents with parental T2DM are a high-risk group for the clustering of cardiometabolic abnormalities and are the primary target to be intervened with a healthy lifestyle. 

Consistent with our findings, a previous study revealed the detrimental effect of a low level of PA on cardiometabolic disorders [13]. A recent study suggested that, independent of PA levels, screen-based sedentary behavior is an emerging risk factor for adiposity and cardiometabolic diseases among children and youth [40]. In a 12-year follow-up study, a higher ST in childhood was observed to be associated with a higher level of BMI, triglycerides, and MetS *Z*-score in early adulthood [14]. In a longitudinal study with multiple life course investigations, an increased number of life periods of frequent TV viewing during adolescence and early adulthood increased the cardiometabolic risk in mid-adulthood [41]. Similarly, our study revealed that adolescents with ≥3 h/day of ST had a 2.2-fold higher risk of MetS; however, an increase of 60.3% in the risk was explained by the child BMI. In a prospective study with mediation analysis, low PA was identified to partially mediate the adverse effect of ST on adiposity, and adiposity was observed to strongly mediate the association between ST and the total-to-HDL-C ratio (a cardiometabolic marker) [42]. ST is a modifiable lifestyle factor, which may promote PA- and adiposity-mediated effects on the cardiometabolic risk. Directing public health efforts toward reducing this sedentary behavior is a crucial concern in adolescent cardiometabolic health care.

Our study revealed that adolescents who consumed >500 mL/day of SSB had elevated parental risk factors and child BMI- and total calorie intake-adjusted MetS risk (aOR = 15.2 and 22.5, respectively, compared with adolescents with pot-MetS and non-MetS adolescents, Table 4). The association between a high consumption of SSB and the development of hypertension, dyslipidemia, dysglycemia, insulin resistance, and hyperuricemia have been reported in recent epidemiological studies in adolescents [4,24,43,44,45,46]. Despite specific controversies, some mechanisms have been proposed to support the argument that excess sugar consumption increases cardiometabolic risk. The direct pathway is associated with disordered hepatic uptake and unregulated fructose metabolism, and the indirect pathway is associated with weight gain and fat accumulation [47]. Moreover, this study revealed that the risk of pot-MetS + MetS associated with SSB consumption was multiplicatively enhanced among adolescents with overweight/obesity (*p* for multiplicative interaction < 0.05), supporting the hypothesis that weight gain is associated with the development and aggregation of cardiometabolic risk factors in a mechanistic manner and thus increasing the effect of added sugar in beverages.

Passive smoking has been associated with an increased risk of MetS and a few cardiometabolic risk factors among adolescents [48,49]. In the 2007–2010 National Health and Nutrition Examination Survey, the joint risk of adolescent MetS from high exposure to passive smoking and low levels of vitamin E or omega-3 polyunsaturated fatty acids was higher than that expected from the individual risks [50]. However, in this study, no significant effects of parental smoking on adolescent MetS were identified.

This study has several strengths. First, according to our review of relevant literature, this study is the first to evaluate the associations of parental and pediatric risk factors with the clustering of cardiometabolic risk factors among Taiwanese adolescents; moreover, a large representative data set was used. Second, because highly trained staff members cautiously explained the data filling protocol and because participants were unaware of the research hypothesis, reporting bias should be limited; even if it exists, the association measure should be biased toward the null. However, this study has several limitations. First, although anthropometric measurements and lifestyle factors were obtained three weeks before collecting clinical blood samples, the cross-sectional study design prevents any cause–effect determination. Second, parental blood samples were not collected, thus limiting the valuation of familial aggregation for all MetS components. Finally, participants with pot-MetS and MetS were combined because of the low prevalence of MetS, thus limiting interaction assessments between risk factors for adolescents with MetS.

## 5. Conclusions

Parental overweight and cardiometabolic diseases and pediatric sedentary and high sugar-intake lifestyles correlate with the development of adolescent MetS, and an elevated child BMI explains a part of these associations. Pediatric adiposity might be multiplicatively associated with SSB consumption for enhancing the MetS prevalence ratio among adolescents.

## Figures and Tables

**Table 1:** Demographic backgrounds and risk factors of adolescents in relation to metabolic syndrome status.

Factors	Non-MetS	Pot-MetS	Diff.1 ^1^	MetS	Diff.2 ^1^	*p* Value ^2^
**Study number**	1565	1077		85		
**Design-adjusted distribution ^3^**						
**Prevalence pattern, %**	57.4	39.3		3.3		<0.001
**Demographic and risk factor**						
**Age (year), mean ± SE**	13.6 ± 0.1	13.5 ± 0.1	−0.1	13.6 ± 0.1	−0.1	0.454
**Male, %**	49.7	46.1	−3.6	64.1	14.4 *	0.027
**Ethnicity, %**						
Fukienese	67.3	70.6		67.2		0.214
Hakka	10.6	7.3		11.9		
Aboriginal	3.1	4.6		3.5		
Others	19.0	17.5		17.3		
**Residential area, %**						
Kaohsiung	61.4	52.6		51.3		0.300
Pingtung	31.3	37.7		39.7		
Taitung	7.3	9.8		9.0		
**Total calorie intake (kcal/d), mean ± SE**	2092.9 ± 27.6	2050.5 ± 49.8	−42.4	2127.3 ± 53.0	34.4	0.449
**Physical activity (MET·min/week), mean ± SE**	2474.6 ± 99.3	2409.4 ± 125.1	−65.2	1693.1 ± 179.8	-781.5 *	0.001
**SSB intake (mL/day), mean ± SE**	446.3 ± 10.7	478.3 ± 12.9	32.0 *	562.2 ± 22.2	115.9 *	<0.001
**Alcohol drinking, %**	13.0	9.3	−3.7	10.5	−2.5	0.108
**Cigarette smoking, %**	2.2	2.7	0.5	1.2	−1.0	0.638
**Central obesity, %**	0.0	28.3	28.3 *	89.9	89.9*	<0.001
**zBMI, mean ± SE ^4^**	−0.3 ± 0.03	0.3 ± 0.07	0.6 *	1.9 ± 0.13	2.2 *	<0.001
**MetS component, mean ± SE**						
Waist circumference (cm)	68.8 ± 0.4	75.5 ± 1.2	6.7 *	94.7 ± 1.6	25.9 *	<0.001
Systolic blood pressure (mmHg)	106.4 ± 0.5	112.7 ± 0.7	6.3 *	130.6 ± 2.1	24.1 *	<0.001
Diastolic blood pressure (mmHg)	63.4 ± 0.3	66.5 ± 0.5	3.1 *	74.0 ± 1.7	10.5 *	<0.001
Serum triglyceride (mg/dL)	68.5 ± 0.8	86.9 ± 3.2	18.4 *	138.3 ± 9.0	69.8 *	<0.001
Serum high-density lipoprotein cholesterol (mg/dL)	61.7 ± 0.6	51.8 ± 2.2	−10.0 *	45.6 ± 1.9	−16.1 *	<0.001
Fasting plasma glucose (mg/dL)	89.5 ± 0.4	92.8 ± 1.5	3.2 *	97.9 ± 1.7	8.4 *	<0.001

Abbreviations: non-MetS, non-metabolic syndrome; pot-MetS, potential metabolic syndrome; MetS, metabolic syndrome. Adolescents with 0, 1–2 and ≥3 risk components for MetS were defined as non-MetS, pot-MetS and MetS, respectively; * *p* < 0.05. ^1^ Diff.1 and Diff.2 denote the difference in mean or proportion for pot-MetS vs. non-MetS and MetS vs. non-MetS, respectively; ^2^ *p* values for mean or percentage differences across MetS groups; ^3^ Data was adjusted for sample weight and the complex study design; ^4^ zBMI denotes an age-sex-standardized *z*-score for BMI value.

**Table 2:** Adjusted odds ratios of adolescent potential metabolic syndrome and metabolic syndrome associated with parental risk factors.

Factors	Non-MetS	Pot-MetS			MetS			MetS vs. Pot-MetS	
	%	%	aOR ^1^	(95% CI)	%	aOR ^1^	(95% CI)	aOR Ratio ^1^	(95% CI)
**Parental bodyweight**									
**Father**									
NW	45.9	35.6	1.0		28.5	1.0		1.0	
OW + OB	54.1	64.4	1.5	(1.3–1.8)	71.5	2.0	(0.9–4.4)	1.3	(0.6–3.0)
**Mother**									
NW	73.1	63.1	1.0		54.3	1.0		1.0	
OW + OB	26.9	36.9	1.5	(1.2–1.9)	45.7	2.2	(1.2–4.1)	1.4	(0.8–2.5)
**Father and mother**									
Both NW	35.2	25.3	1.0		20.9				
One OW + OB	49.2	48.0	1.4	(1.0–1.9)	46.2	1.5	(0.5–4.6)	1.1	(0.4–3.2)
Both OW + OB	15.7	26.6	2.3	(1.6–3.3)	32.9	3.2	(1.3–8.1)	1.4	(0.6–3.4)
**Parental disease**									
**Diabetes mellitus**									
No	95.7	93.2	1.0		81.0	1.0		1.0	
Yes	4.3	6.8	1.7	(1.1–2.7)	19.0	5.1	(2.7–9.7)	3.0	(1.3–6.8)
**Hypertension**									
No	87.4	82.3	1.0		72.0	1.0		1.0	
Yes	12.6	17.7	1.5	(1.1–2.0)	28.0	2.7	(1.4–5.3)	1.8	(1.1–3.1)
**Dyslipidemia**									
No	86.9	87.6	1.0		81.1	1.0		1.0	
Yes	13.1	12.4	1.0	(0.7–1.3)	18.9	1.5	(0.7–3.3)	1.6	(0.7–3.3)

Abbreviations: aOR, adjusted odds ratios; NW, normal weight; OB, obesity; OW, overweight; non-MetS, non-metabolic syndrome; pot-MetS, potential metabolic syndrome; MetS, metabolic syndrome. Adolescents with 0, 1–2 and ≥3 risk components for MetS were defined as non-MetS, pot-MetS and MetS, respectively. ^1^ aORs were adjusted for age, gender, ethnicity, residential area, total calorie intake, alcohol drinking and cigarette smoking.

**Table 3:** Adjusted odds ratios and means of adolescent potential metabolic syndrome and metabolic syndrome associated with adolescent bodyweight and lifestyle factors.

Factors	Non-MetS	Pot-MetS			MetS			MetS vs. Pot-MetS	
	%	%	aOR ^1^	(95% CI)	%	aOR ^1^	(95% CI)	aOR Ratio ^1^	(95% CI)
**Bodyweight ^3^**									
Normal	78.2	50.0	1.0		0.3	1.0		1.0	
Overweight + Obesity	21.9	50.0	3.9	(2.6–5.9)	99.7	1461.2	(198.8–10741.8)	373.9	(50.7–2759.0)
Adjusted BMI mean ^1^	19.9	22.9	3.0 ^2,^*		30.3	10.4 ^2,^*		7.4 ^2,^*	
**Physical activity (MET·min/week)**									
≥2140.5	32.5	30.7	1.0		16.4	1.0		1.0	
952.4–2140.4	43.4	41.8	1.0	(0.7–1.3)	45.2	2.5	(1.2–5.0)	2.5	(1.3–5.1)
<952.4	24.1	27.5	1.1	(0.8–1.5)	38.4	4.4	(2.2–8.6)	3.8	(1.9–7.8)
Adjusted mean ^1^	2454.1	2454.2	0.2 ^2^		1514.1	−940.0 ^2,^*		−940.1 ^2,^*	
**Screen time (h/day)**									
<1.5	49.4	45.9	1.0		36.8	1.0		1.0	
1.5–2.9	34.7	37.2	1.2	(0.9–1.6)	38.9	1.5	(0.8–2.8)	1.3	(0.7–2.3)
≥3	15.9	16.9	1.2	(0.9–1.6)	24.3	2.1	(1.1–3.9)	1.8	(0.9–3.5)
Adjusted mean ^1^	1.63	1.70	0.07 ^2^		1.93	0.30 ^2,^*		0.22 ^2^	
**Reading time (h/day)**									
<1.5	47.1	52.3	1.0		56.2	1.0		1.0	
1.5–2.9	43.5	38.5	0.8	(0.7–1.0)	31.3	0.6	(0.4–1.0)	0.8	(0.4–1.3)
≥3	9.4	9.2	1.0	(0.7–1.5)	12.5	1.2	(0.3–4.2)	1.2	(0.3–4.6)
Adjusted mean ^1^	1.51	1.45	−0.06 ^2^		1.36	−0.16 ^2^		−0.10 ^2^	
**Sugar-sweetened beverage intake (mL/day)**									
Non-intake	14.0	10.7	1.0		0.8	1.0		1.0	
1–500	63.3	61.8	1.3	(0.9–1.8)	59.9	16.6	(2.0–140.7)	13.0	(1.6–108.0)
>500	22.7	27.5	1.7	(1.1–2.6)	39.4	30.2	(3.6–250.5)	17.7	(2.1–152.8)
Adjusted mean ^1^	445.2	481.1	35.9 ^2,^*		549.2	104.0 ^2,^*		68.1 ^2,^*	

Abbreviation: aOR, adjusted odds ratios; non-MetS, non-metabolic syndrome; pot-MetS, potential metabolic syndrome; MetS, metabolic syndrome (adolescents with 0, 1–2 and ≥3 risk components for MetS were defined as non-MetS, pot-MetS and MetS, respectively); * *p* < 0.05. ^1^ aORs and adjusted means were adjusted for age, gender, ethnicity, residential area, total calorie intake, alcohol drinking and cigarette smoking; ^2^ Differences in adjusted means between the compared groups (pot-MetS and MetS vs. non-MetS, and MetS vs. pot-MetS); ^3^ Bodyweight was classified according to adolescent age- and sex-specific BMI cut-off points for overweight and obesity determined by Taiwan’s Ministry of Health and Welfare for adolescent growth charts.

**Table 4:** Excess prevalence ratios of adolescent potential metabolic syndrome and metabolic syndrome explained by adolescent body mass index for significant parental and adolescent’s risk factors.

Factors	Base Model ^1,2^			Child zBMI-Adjusted Model ^3^								
	Pot-MetS	MetS	MetS vs. Pot-MetS	Pot-MetS		EPRE% ^4^	MetS		EPRE% ^4^	MetS vs. Pot-MetS		EPRE% ^4^
	aOR	aOR	aOR	aOR	(95% CI)		aOR	(95% CI)		aOR	(95% CI)	
**Parental risk factors**												
Father: OW + OB vs. NW	1.5 *	2.0	1.3	1.1	(0.9–1.4)	72.9	1.0	(0.4–2.4)	na	0.9	(0.4–2.2)	na
Mother: OW + OB vs. NW	1.5 *	1.9 *	1.3	1.2	(0.9–1.4)	66.9	1.0	(0.4–2.7)	95.6	0.8	(0.3–1.7)	na
Diabetes mellitus: Yes vs. No	1.6 *	4.2 *	2.6 *	1.4	(0.8–2.3)	40.1	2.6 *	(1.0–6.5)	50.5	1.9	(0.7–5.0)	44.4
Hypertension: Yes vs. No	1.5 *	2.7 *	1.8 *	1.4	(0.99–1.9)	23.5	2.4 *	(1.2–4.6)	18.8	1.7	(0.99–3.1)	10.2
**Adolescent lifestyle factors**												
Physical activity (MET·min/week)												
952.4–2140.4 vs. ≥2140.5	1.1	3.2 *	2.9 *	1.2	(0.8–1.6)	na	4.6 *	(1.5–14.5)	NA	4.0 *	(1.2–12.8)	NA
<952.4 vs. ≥2140.5	1.3	3.3 *	2.6 *	1.4	(0.9–2.0)	na	2.6	(0.9–8.0)	30.3	1.9	(0.6–5.9)	41.7
Screen time (h/day)												
1.5–2.9 vs. <1.5	1.2	1.5	1.2	1.3	(0.9–1.8)	na	1.5	(0.5–4.7)	na	1.2	(0.6–2.4)	na
≥3 vs. <1.5	1.4	2.2 *	1.5	1.4	(0.9–2.2)	na	1.5	(0.6–3.6)	60.3	1.0	(0.6–2.9)	na
SSB intake (mL/day)												
1–500 vs. Non-intake	1.3	16.1 *	12.6 *	1.2	(0.9–1.8)	na	18.9 *	(1.7–207.5)	NA	15.2 *	(1.4–163.8)	NA
>500 vs. Non-intake	1.6 *	26.9 *	16.4 *	1.5	(0.9–2.2)	23.6	22.5 *	(1.9–265.4)	16.9	15.2 *	(1.3–183.1)	8.3

Abbreviation: aOR, adjusted odds ratio; BMI, body mass index; EPRE, excess prevalence ratio explained; na, non-appreciated because the aOR obtained in the base model was not significant; NA, non-applicable because zBMI produced a negative effect, i.e., aOR was larger in the zBMI-adjusted models than that in the base models, zBMI did not explain any effect; NW, normal weight; OB, obesity; OW, overweight; SSB, sugar-sweetened beverage; non-MetS, non-metabolic syndrome; pot-MetS, potential metabolic syndrome; MetS, metabolic syndrome (adolescents with 0, 1–2 and ≥3 risk components for MetS were defined as non-MetS, pot-MetS and MetS, respectively); * *p* < 0.05. ^1^ In the base models for parental factors, aORs were adjusted for adolescent age, gender, ethnicity, residential area, total calorie intake, alcohol drinking and cigarette smoking, as well as physical activity, screen time and SSB intake; ^2^ In the base models for adolescent lifestyle factors, aORs were adjusted for adolescent age, gender, ethnicity, residential area, total calorie intake, alcohol drinking and cigarette smoking, as well as parental overweight/obesity, diabetes mellitus and hypertension; ^3^ aORs were obtained from the respective base model and additionally adjusted for child zBMI; ^4^ The excess prevalence ratio of a specific risk factor on pot-MetS and MetS that was explained by the child zBMI. EPRE is defined as (aOR_1_ − aOR_2_)/(aOR_1_ − 1), where aOR_1_ is the prevalence ratio obtained from the base model; aOR_2_ is the prevalence ratio after additionally adjusting for the child zBMI; and aOR_1_ − 1 is the excess prevalence ratio of a risk factor.

**Table 5:** Combined and interaction effects of parental risk factors and adolescent lifestyle factors on adolescent potential metabolic syndrome and metabolic syndrome.

Factor	Non-MetS	Pot-MetS + MetS			Additive Model		Multiplicative Model	
	%	%	aOR ^1^	(95% CI)	SI ^2^	(95% CI)	EOR ^2^	*p*-Value
**Parental risk factors and adolescent bodyweight**								
**Father BW/BW**								
NW/NW	43.2	23.9	1.0					
NW/OW + OB	2.7	11.1	8.1	(4.4–14.9)				
OW + OB/NW	46.6	34.9	1.3	(1.1–1.7)				
OW + OB/OW + OB	7.5	30	7.9	(4.4–14.0)	0.9	(0.5–1.7)	10.5	0.336
**Mother BW/BW**								
NW/NW	66.6	40.9	1.0					
NW/OW + OB	6.6	21.5	6.4	(3.5–11.8)				
OW + OB/NW	23.5	18.8	1.3	(0.9–1.7)				
OW + OB/OW + OB	3.4	18.8	9.6	(5.0–18.2)	1.5	(0.8–2.8)	8.3	0.641
**Diabetes/BW**								
No/NW	85.5	56.1	1.0					
No/OW + OB	10.2	36.2	6.2	(3.6–10.7)				
Yes/NW	4.0	2.8	1.2	(0.6–2.2)				
Yes/OW + OB	0.3	5	23.8	(8.3–68.5)	4.2	(1.4–12.4)	7.4	0.08
**Hypertension/BW**								
No/NW	78.2	49.2	1.0					
No/OW + OB	9.2	32.4	6.5	(3.8–10.8)				
Yes/NW	11.3	9.7	1.4	(0.9–2.0)				
Yes/OW + OB	1.4	8.7	11.2	(4.6–27.4)	1.7	(0.9–3.5)	9.1	0.477
**Adolescent lifestyle factor and bodyweight**								
**Physical activity (MET·min/week)/BW**								
≥952.4/NW	28.0	16.5	1.0					
≥952.4/OW + OB	4.5	13.2	5.6	(3.0–10.5)				
<952.4/NW	61.5	42.4	1.1	(0.8–1.4)				
<952.4/OW + OB	6.0	28.0	8.0	(4.6–13.9)	1.5	(0.8–2.7)	6.2	0.298
**Screen time (h/day)/BW**								
<1.5/NW	44.0	29.1	1.0					
<1.5/OW + OB	5.4	16.2	5.2	(2.7–10.0)				
≥1.5/NW	45.5	29.8	1.0	(0.7–1.5)				
≥1.5/OW + OB	5.1	25.0	8.8	(4.8–16.4)	1.8	(1.2–2.9)	5.2	0.067
**SSB intake/BW**								
No/NW	12.3	7.5	1.0					
No/OW + OB	1.8	2.4	2.6	(1.0–6.5)				
Yes/NW	77.2	51.4	1.1	(0.8–1.7)				
Yes/OW + OB	8.8	38.7	8.6	(4.3–17.3)	4.4	(1.6–12.6)	2.9	0.009

Abbreviation: BW, bodyweight; NW, normal weight; OB, obesity; OW, overweight; SSB, sugar-sweetened beverage. non-MetS, non-metabolic syndrome; pot-MetS, potential metabolic syndrome; MetS, metabolic syndrome. Adolescents with 0, 1–2 and ≥3 risk components for MetS were defined as non-MetS, pot-MetS and MetS, respectively. ^1^ aORs were adjusted for age, gender, ethnicity, residential area, total calorie intake, alcohol drinking and cigarette smoking; ^2^ Synergism index (SI) was estimated by additive interaction models. Expected odds ratio (EOR) was estimated by multiplicative interaction models.

## Supplementary Materials

The following are available online at http://www.mdpi.com/2072-6643/8/9/567/s1, Table S1: Combined and interaction effects of adolescents’ lifestyle factors on potential metabolic syndrome and metabolic syndrome. Figure S1: Distribution of overweight and obesity among adolescents with non-metabolic syndrome (non-MetS), potential metabolic syndrome (pot-MetS) and metabolic syndrome (MetS).

## Abbreviations

The following abbreviations are used in this manuscript:

aOR	adjusted odds ratio
BMI	body mass index
BP	blood pressure
CI	confidence interval
HDL-C	high-density lipoprotein cholesterol
MET	metabolic equivalent task
MetS	metabolic syndrome
non-MetS	non-metabolic syndrome
PA	physical activity
pot-MetS	potential metabolic syndrome
SI	synergism index
SSB	sugar-sweetened beverage
ST	screen time
T2DM	type 2 diabetes mellitus
