# Supplementary material for: Association of Parental Overweight and Cardiometabolic Diseases and Pediatric Adiposity and Lifestyle Factors with Cardiovascular Risk Factor Clustering in Adolescents
Source: Nutrients. 2016 Sep 13;8(9):567. doi: 10.3390/nu8090567 (PMC5037552; doi:10.3390/nu8090567)
Supplement: Supplementary file 1 [file nutrients-08-00567-s001.docx]

Supplementary Materials: Association of Parental Overweight and Cardiometabolic Diseases and Pediatric Adiposity and Lifestyle Factors with Cardiovascular Risk Factor Clustering in Adolescents

Chun-Ying Lee, Wei-Ting Lin, Sharon Tsai, Yu-Chan Hung, Pei-Wen Wu, Yu-Cheng Yang,
Te-Fu Chan, Hsiao-Ling Huang, Yao-Lin Weng, Yu-Wen Chiu, Chia-Tsuan Huang
and Chien-Hung Lee

**Table S1.** Combined and interaction effects of adolescents’ lifestyle factors on potential metabolic syndrome and metabolic syndrome.

| **Factor** | **Non-MetS** | **Pot-MetS + MetS** | | | **Additive Model** | | **Multiplicative Model** | |
| --- | --- | --- | --- | --- | --- | --- | --- | --- |
|  | **%** | **%** | **aOR ^1^** | **(95% CI)** | **SI ^2^** | **(95% CI)** | **EOR ^2^** | ***p*-Value** |
| **Screen time/Physical activity** |  |  | | |  | |  |  |
| <1.5/≥952.4 | 15.3 | 12.8 | 1.0 |  |  |  |  |  |
| <1.5/<952.4 | 34.1 | 32.4 | 1.1 | (0.8–1.5) |  |  |  |  |
| ≥1.5/≥952.4 | 17.2 | 16.8 | 1.2 | (0.8–1.9) |  |  |  |  |
| ≥1.5/<952.4 | 33.4 | 38.0 | 1.3 | (0.9–1.9) | 0.8 | (0.2–2.7) | 1.3 | 0.981 |
| **SSB intake/ Physical activity** |  |  | | |  | |  |  |
| No/≥952.4 | 3.2 | 1.8 | 1.0 |  |  |  |  |  |
| No/<952.4 | 10.8 | 8.1 | 1.3 | (0.6–2.7) |  |  |  |  |
| Yes/≥952.4 | 29.3 | 27.8 | 1.7 | (0.9–3.1) |  |  |  |  |
| Yes/<952.4 | 56.7 | 62.3 | 1.8 | (0.9–3.5) | 0.8 | (0.4–1.8) | 2.2 | 0.709 |
| **Screen time/SSB intake** |  |  | | |  | |  |  |
| <1.5/No | 10.5 | 7.3 | 1.0 |  |  |  |  |  |
| <1.5/Yes | 39.0 | 37.9 | 1.4 | (0.9–2.1) |  |  |  |  |
| ≥1.5/No | 3.6 | 2.6 | 1.1 | (0.7–1.9) |  |  |  |  |
| ≥1.5/Yes | 47.0 | 52.2 | 1.6 | (1.0–2.6) | 0.9 | (0.3–3.0) | 1.5 | 0.897 |

Abbreviations: non-MetS, non-metabolic syndrome; pot-MetS, potential metabolic syndrome; MetS, metabolic syndrome. Adolescents with 0, 1–2 and ≥3 risk components for MetS were defined as non-MetS, pot-MetS and MetS, respectively. Physical activity was measured in MET∙min/week; Screen time was measured in hour/day; SSB: sugar-sweetened beverage. ^1^ aORs were adjusted for age, gender, ethnicity, residential area, total calorie intake, alcohol drinking and cigarette smoking;  ^2^ Synergism index (SI) was estimated by additive interaction models. Expected odds ratio (EOR) was estimated by multiplicative interaction models.

**Figure S1.** Distribution of overweight and obesity among adolescents with non-metabolic syndrome (non-MetS), potential metabolic syndrome (pot-MetS) and metabolic syndrome (MetS). Note: Normal weight, overweight and obesity were defined according to the age- and sex-specific criteria determined by Taiwan’s Ministry of Health and Welfare for adolescent growth charts. Adolescents with 0, 1–2 and ≥3 risk components for MetS were defined as non-MetS, pot-MetS and MetS, respectively.
